# Bis[μ-4-(1*H*-imidazol-3-ium-1-yl)benzoato-κ^2^ *O*:*O*′]bis­[(methanol)tris­(nitrato-κ^2^ *O*,*O*′)terbium(III)]

**DOI:** 10.1107/S1600536811002443

**Published:** 2011-01-22

**Authors:** Yeong-Min Jung, Soon W. Lee

**Affiliations:** a Department of Chemistry (BK21), Sungkyunkwan University, Natural Science Campus, Suwon 440-746, Republic of Korea

## Abstract

In the centrosymmetric dinuclear title complex, [Tb_2_(NO_3_)_6_(C_10_H_8_N_2_O_2_)_2_(CH_3_OH)_2_], the Tb atoms are bridged by the carboxyl­ate groups of the two 4-(1*H*-imidazol-3-ium-1-yl)benzoate (iba) ligands. The iba ligand adopts a zwitterionic form with a protonated imidazole group. The Tb atom adopts a distorted tricapped trigonal–prismatic coordination geometry and is coordinated by six O atoms of three chelating nitrate ions, one O atom of the methanol mol­ecule and two O atoms of two iba ligands. The intra­molecular Tb⋯Tb separation is 5.1419 (3) Å. O—H⋯O and N—H⋯O hydrogen bonds connect complex mol­ecules into a two-dimensional network.

## Related literature

For the preparation of 4-(1*H*-imidazol-1-yl)benzoic acid (iba), see: Zhang *et al.* (2007**a* ▶)*. To the best of our knowledge, the title compound is the first *f*-block complex of the iba ligand. For *d*-block coordination compounds of the iba ligand, see: Aijaz *et al.* (2009 ▶); Bai *et al.* (2009 ▶); Gao *et al.* (2008 ▶, 2009 ▶); Niu *et al.* (2009 ▶); Zhang *et al.* (2007**a* ▶,*b* ▶)*.
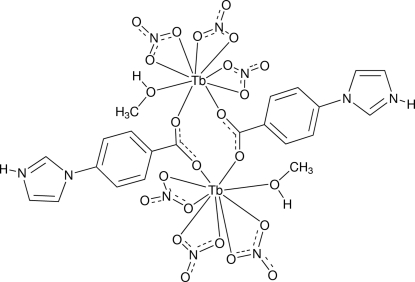


## Experimental

### 

#### Crystal data

[Tb_2_(NO_3_)_6_(C_10_H_8_N_2_O_2_)_2_(CH_4_O)_2_]
*M* *_r_* = 1130.35
Triclinic, 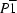

*a* = 8.2966 (3) Å
*b* = 9.6107 (3) Å
*c* = 11.6780 (4) Å
α = 95.328 (2)°
β = 101.271 (2)°
γ = 100.166 (2)°
*V* = 891.01 (5) Å^3^
*Z* = 1
Mo *K*α radiation
μ = 4.05 mm^−1^
*T* = 296 K
0.32 × 0.24 × 0.20 mm

#### Data collection

Bruker SMART CCD area-detector diffractometer
Absorption correction: multi-scan (*SADABS*; Sheldrick, 1996 ▶) *T*_min_ = 0.358, *T*_max_ = 0.498
14627 measured reflections
4356 independent reflections
3924 reflections with *I* > 2σ(*I*)
*R*_int_ = 0.037

#### Refinement

*R*[*F* ^2^ > 2σ(*F* ^2^)] = 0.026
*wR*(*F* ^2^) = 0.061
*S* = 1.04
4356 reflections
266 parameters
H atoms treated by a mixture of independent and constrained refinement
Δρ_max_ = 1.01 e Å^−3^
Δρ_min_ = −0.49 e Å^−3^

### 

Data collection: *SMART* (Bruker, 1997 ▶); cell refinement: *SAINT* (Bruker, 1997 ▶); data reduction: *SAINT*; program(s) used to solve structure: *SHELXTL* (Sheldrick, 2008 ▶); program(s) used to refine structure: *SHELXTL*; molecular graphics: *SHELXTL*; software used to prepare material for publication: *SHELXTL*.

## Supplementary Material

Crystal structure: contains datablocks I, global. DOI: 10.1107/S1600536811002443/gk2337sup1.cif

Structure factors: contains datablocks I. DOI: 10.1107/S1600536811002443/gk2337Isup2.hkl

Additional supplementary materials:  crystallographic information; 3D view; checkCIF report

## Figures and Tables

**Table 1:** Selected bond lengths (Å)

Tb1—O2^i^	2.245 (2)
Tb1—O1	2.275 (2)
Tb1—O12	2.385 (3)
Tb1—O3	2.441 (3)
Tb1—O6	2.461 (2)
Tb1—O4	2.464 (3)
Tb1—O10	2.509 (3)
Tb1—O7	2.529 (3)
Tb1—O9	2.553 (3)

**Table 2:** Hydrogen-bond geometry (Å, °)

*D*—H⋯*A*	*D*—H	H⋯*A*	*D*⋯*A*	*D*—H⋯*A*
O12—H1*O*⋯O10^ii^	0.81	2.15	2.960 (4)	173
N2—H2*N*⋯O6^iii^	0.80 (5)	2.00 (5)	2.779 (4)	167 (5)

Symmetry codes: (ii) 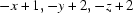; (iii) 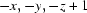.

**Fig. 1.**
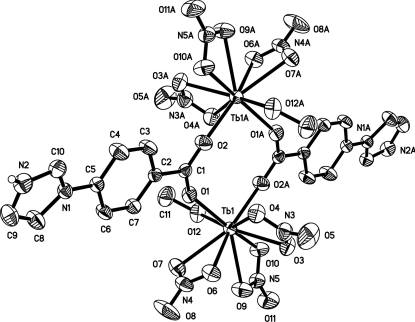
Molecular structure of the title complex showing 50% probability displacement ellipsoids. Symmetry code for the atoms with A in their labels: -x + 1, -y + 1, -z + 2.

**Fig. 2.**
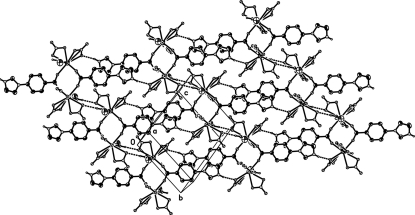
A two dimensional network formed by N—H···O and O—H···O hydrogen bonds.

## supplementary crystallographic information

### Comment

4-(1*H*-Imidazol-1-yl)benzoic acid (iba) is a potential bridging ligand possessing two coordinating groups: a carboxylate group and an imidazole group. This ligand was originally prepared by Chen's group (Zhang *et al.*, 2007*a*). The iba ligand was recently employed to prepare a couple of discrete complexes of *d*-block metals such as Mn (Gao *et al.*, 2008) and Ni (Zhang *et al.*, 2007*b*). It was also utilized for the preparation of coordination polymers of *d*-block metals such as Co (Zhang *et al.*, 2007*a*), Cu (Niu, *et al.*, 2009), Zn (Bai *et al.*, 2009), and Cd (Aijaz *et al.*, 2009; Bai *et al.*, 2009; Gao *et al.*, 2009). By contrast, the corresponding *f*-block complexes, discrete or polymeric, are not known at present. In this context, we attempted to prepare *f*-block coordination polymers by using the iba ligand. Inconsistent with our expectation, however, a discrete molecular species (the title complex) was produced. We herein report the preparation and structure of a terbium complex, the first *f*-block complex of the iba ligand.

The local coordination environment of the Tb^III^ ion in the title complex is presented in Fig. 1, which shows two iba ligands linking two 9-coordinate Tb^III^ ions. The asymmetric unit consists of only half the formula unit, and the other half is generated by crystallographic inversion center. All atoms occupy general positions, and the inversion point is located at the center of the title complex. The imidazole nitrogen (N2) in the iba ligand remains uncoordinated and is protonated. As a result, the iba ligand has a net charge of zero and acts as a zwitterion in which a positive charge is on the imidazole N atom and a negative charge is on the carboxylate group. It would be meaningful to notice that the imidazole N atom is coordinated to the metals in all known *d*-block metal complexes and coordination polymers. Additional coordination of the iba carboxylate group to the metals led to the formation of 2-D or 3-D coordination polymers (Zhang *et al.*, 2007*a*; Niu, *et al.*, 2009; Bai *et al.*, 2009; Aijaz *et al.*, 2009; Bai *et al.*, 2009; Gao *et al.*, 2009). The fact that the N2 atom is not bonded to the Tb^III^ ion in the title complex can probably be explained on the basis of the hard–soft acid–base concept. The hard Tb^III^ ion is expected to have a preference to coordinating to the harder oxygen atom (in the carboxylate group) over the softer nitrogen atom (in the imidazole group).

Each Tb^III^ ion is coordinated to six O atoms from three NO_3_^-^, one O atom from CH_3_OH, and two O atoms from two bis(monodentate) iba ligands. The [TbO_9_] core forms a distorted tricapped trigonal prism. Two Tb^III^ ions and two carboxylate groups form a central eight-membered ring. All nitrate ligands act as bidentate ligands. The Tb···Tb separation is 5.1419 (3) Å, which is much longer than the sum (4.0 Å) of van der Waals radii of the two Tb^III^ ions and therefore rules out direct interaction between the two metal ions. The N–H of the imidazole group and O–H of the coordinated methanol molecule participate in the intermolecular hydrogen bonds, which connect the molecules of the title complexe into a two-dimensional network (Fig. 2).

### Experimental

A mixture of Tb(NO_3_)_3_.5H_2_O (87 mg, 0.2 mmol), 4-(1*H*-imidazol-1-yl)benzoic acid (37 mg, 0.2 mmol), and CH_3_OH (6 ml) was heated at 343 K for 24 h in a 23 ml Teflon-lined stainless-steel autoclave and then cooled slowly to room temperature for 24 h. The resulting colorless crystals were collected by filtration, washed by ethanol (5 ml × 3) and dichloromethane (5 ml × 3), and then air-dried to give the title complex (82 mg, 0.072 mmol, 37%). mp: 542–544 K. IR (KBr, cm^-1^): 3889 (w), 3826 (w), 3751 (w), 2912 (*m*), 2628 (*m*), 2364 (*m*), 2069 (*m*), 1792 (*m*), 1591 (*m*), 943 (*m*).

### Refinement

The H atom of the methanol OH group was generated in the idealized position [O-H = 0.81Å, *U*_iso_(H) = 1.5U_eq_(O)] and refined in a riding model approximation. The NH hydrogen atom was located in difference Fourier maps and freely refined. The remaining H atoms were generated in idealized positions (C–H = 0.93–0.96 Å) and refined as riding with *U*_iso_(H) = 1.2U_eq_(C).

### Crystal data

**Table:**

[Tb_2_(NO_3_)_6_(C_10_H_8_N_2_O_2_)_2_(CH_4_O)_2_]	*Z* = 1
*M**_r_* = 1130.35	*F*(000) = 548
Triclinic, *P*1	*D*_x_ = 2.107 Mg m^−^^3^
Hall symbol: -P 1	Mo *K*α radiation, λ = 0.71073 Å
*a* = 8.2966 (3) Å	Cell parameters from 9939 reflections
*b* = 9.6107 (3) Å	θ = 2.7–28.5°
*c* = 11.6780 (4) Å	µ = 4.05 mm^−^^1^
α = 95.328 (2)°	*T* = 296 K
β = 101.271 (2)°	Block, colourless
γ = 100.166 (2)°	0.32 × 0.24 × 0.20 mm
*V* = 891.01 (5) Å^3^	

### Data collection

**Table:**

Bruker SMART CCD area-detector diffractometer	4356 independent reflections
Radiation source: sealed tube	3924 reflections with *I* > 2σ(*I*)
graphite	*R*_int_ = 0.037
φ and ω scans	θ_max_ = 28.5°, θ_min_ = 2.2°
Absorption correction: multi-scan (*SADABS*; Sheldrick, 1996)	*h* = −11→11
*T*_min_ = 0.358, *T*_max_ = 0.498	*k* = −12→12
14627 measured reflections	*l* = −15→15

### Refinement

**Table:**

Refinement on *F*^2^	Primary atom site location: structure-invariant direct methods
Least-squares matrix: full	Secondary atom site location: difference Fourier map
*R*[*F*^2^ > 2σ(*F*^2^)] = 0.026	Hydrogen site location: inferred from neighbouring sites
*wR*(*F*^2^) = 0.061	H atoms treated by a mixture of independent and constrained refinement
*S* = 1.04	*w* = 1/[σ^2^(*F*_o_^2^) + (0.0361*P*)^2^] where *P* = (*F*_o_^2^ + 2*F*_c_^2^)/3
4356 reflections	(Δ/σ)_max_ = 0.002
266 parameters	Δρ_max_ = 1.01 e Å^−^^3^
0 restraints	Δρ_min_ = −0.49 e Å^−^^3^

### Special details

**Table:**

Geometry. All e.s.d.'s (except the e.s.d. in the dihedral angle between two l.s. planes) are estimated using the full covariance matrix. The cell e.s.d.'s are taken into account individually in the estimation of e.s.d.'s in distances, angles and torsion angles; correlations between e.s.d.'s in cell parameters are only used when they are defined by crystal symmetry. An approximate (isotropic) treatment of cell e.s.d.'s is used for estimating e.s.d.'s involving l.s. planes.
Refinement. Refinement of *F*^2^ against ALL reflections. The weighted *R*-factor *wR* and goodness of fit *S* are based on *F*^2^, conventional *R*-factors *R* are based on *F*, with *F* set to zero for negative *F*^2^. The threshold expression of *F*^2^ > σ(*F*^2^) is used only for calculating *R*-factors(gt) *etc*. and is not relevant to the choice of reflections for refinement. *R*-factors based on *F*^2^ are statistically about twice as large as those based on *F*, and *R*- factors based on ALL data will be even larger.

### Fractional atomic coordinates and isotropic or equivalent isotropic displacement parameters (Å^2^)

**Table:**

	*x*	*y*	*z*	*U*_iso_*/*U*_eq_	
Tb1	0.457118 (19)	0.675119 (14)	0.848450 (11)	0.03284 (6)	
O1	0.2845 (3)	0.4704 (2)	0.8671 (2)	0.0460 (6)	
O2	0.3792 (4)	0.3116 (3)	0.9719 (2)	0.0512 (6)	
O3	0.7282 (4)	0.6892 (3)	0.7935 (3)	0.0581 (7)	
O4	0.5994 (4)	0.4789 (3)	0.8034 (3)	0.0553 (7)	
O5	0.8288 (6)	0.5102 (5)	0.7405 (4)	0.1010 (14)	
O6	0.3662 (4)	0.5789 (3)	0.6383 (2)	0.0515 (6)	
O7	0.1885 (4)	0.6835 (3)	0.7055 (3)	0.0592 (7)	
O8	0.1450 (6)	0.6100 (4)	0.5188 (3)	0.1017 (15)	
O9	0.4888 (5)	0.8709 (3)	0.7163 (2)	0.0649 (8)	
O10	0.5819 (4)	0.9363 (3)	0.9002 (2)	0.0550 (7)	
O11	0.6051 (5)	1.0899 (3)	0.7783 (3)	0.0784 (10)	
O12	0.2864 (4)	0.7787 (3)	0.9605 (2)	0.0573 (7)	
H1O	0.3305	0.8559	0.9989	0.086*	
N1	−0.1852 (3)	−0.0986 (3)	0.5742 (2)	0.0356 (5)	
N2	−0.3091 (5)	−0.3070 (3)	0.4848 (3)	0.0497 (8)	
N3	0.7211 (5)	0.5573 (4)	0.7785 (3)	0.0555 (8)	
N4	0.2299 (5)	0.6250 (3)	0.6183 (3)	0.0523 (8)	
N5	0.5595 (4)	0.9700 (3)	0.7973 (3)	0.0472 (7)	
C1	0.2827 (4)	0.3458 (3)	0.8879 (3)	0.0344 (6)	
C2	0.1576 (4)	0.2281 (3)	0.8068 (3)	0.0326 (6)	
C3	0.1500 (5)	0.0880 (4)	0.8264 (3)	0.0488 (9)	
H3	0.2213	0.0662	0.8912	0.059*	
C4	0.0369 (6)	−0.0201 (4)	0.7502 (3)	0.0538 (10)	
H4	0.0328	−0.1145	0.7632	0.065*	
C5	−0.0694 (4)	0.0123 (3)	0.6554 (3)	0.0332 (6)	
C6	−0.0660 (5)	0.1519 (4)	0.6361 (3)	0.0446 (8)	
H6	−0.1396	0.1735	0.5724	0.054*	
C7	0.0478 (4)	0.2594 (3)	0.7121 (3)	0.0409 (7)	
H7	0.0506	0.3538	0.6995	0.049*	
C8	−0.3167 (5)	−0.0831 (4)	0.4857 (3)	0.0526 (9)	
H8	−0.3467	0.0022	0.4670	0.063*	
C9	−0.3922 (5)	−0.2143 (5)	0.4321 (3)	0.0554 (10)	
H9	−0.4852	−0.2370	0.3698	0.066*	
C10	−0.1834 (5)	−0.2376 (4)	0.5700 (3)	0.0434 (7)	
H10	−0.1070	−0.2784	0.6186	0.052*	
C11	0.1366 (7)	0.7167 (6)	0.9925 (5)	0.0750 (14)	
H11A	0.0994	0.7879	1.0384	0.113*	
H11B	0.1569	0.6410	1.0381	0.113*	
H11C	0.0518	0.6792	0.9227	0.113*	
H2N	−0.325 (6)	−0.389 (6)	0.460 (4)	0.064 (14)*	

### Atomic displacement parameters (Å^2^)

**Table:**

	*U*^11^	*U*^22^	*U*^33^	*U*^12^	*U*^13^	*U*^23^
Tb1	0.03738 (9)	0.02521 (8)	0.03241 (8)	0.00405 (6)	0.00267 (5)	0.00046 (5)
O1	0.0431 (14)	0.0275 (11)	0.0608 (14)	−0.0002 (10)	0.0031 (11)	0.0038 (10)
O2	0.0586 (17)	0.0418 (13)	0.0407 (12)	−0.0013 (12)	−0.0077 (11)	0.0037 (10)
O3	0.0486 (16)	0.0517 (16)	0.0746 (18)	0.0049 (13)	0.0199 (13)	0.0076 (13)
O4	0.0537 (17)	0.0403 (14)	0.0748 (17)	0.0140 (13)	0.0172 (13)	0.0072 (12)
O5	0.093 (3)	0.098 (3)	0.148 (4)	0.056 (3)	0.072 (3)	0.029 (3)
O6	0.0658 (18)	0.0396 (13)	0.0424 (12)	0.0117 (12)	0.0008 (11)	−0.0071 (10)
O7	0.0545 (17)	0.0576 (17)	0.0631 (17)	0.0208 (14)	−0.0009 (13)	0.0064 (13)
O8	0.128 (4)	0.090 (3)	0.0587 (19)	0.019 (3)	−0.045 (2)	0.0059 (18)
O9	0.096 (2)	0.0408 (14)	0.0464 (14)	0.0011 (15)	−0.0020 (14)	0.0055 (11)
O10	0.082 (2)	0.0377 (13)	0.0405 (12)	0.0010 (13)	0.0129 (12)	0.0018 (10)
O11	0.105 (3)	0.0331 (14)	0.088 (2)	−0.0013 (16)	0.007 (2)	0.0200 (14)
O12	0.0515 (16)	0.0553 (16)	0.0598 (15)	0.0053 (13)	0.0147 (12)	−0.0152 (12)
N1	0.0358 (14)	0.0319 (13)	0.0357 (12)	0.0046 (11)	0.0036 (10)	0.0007 (10)
N2	0.058 (2)	0.0338 (15)	0.0493 (16)	−0.0016 (14)	0.0080 (14)	−0.0071 (12)
N3	0.049 (2)	0.062 (2)	0.0601 (19)	0.0181 (17)	0.0164 (15)	0.0073 (16)
N4	0.063 (2)	0.0357 (15)	0.0472 (16)	0.0061 (15)	−0.0106 (15)	0.0071 (12)
N5	0.0543 (19)	0.0322 (14)	0.0536 (17)	0.0057 (13)	0.0103 (14)	0.0056 (12)
C1	0.0349 (16)	0.0320 (15)	0.0330 (14)	0.0000 (12)	0.0068 (12)	0.0010 (11)
C2	0.0350 (16)	0.0283 (14)	0.0334 (13)	0.0037 (12)	0.0073 (11)	0.0033 (10)
C3	0.059 (2)	0.0328 (16)	0.0434 (17)	0.0047 (16)	−0.0126 (15)	0.0060 (13)
C4	0.068 (3)	0.0260 (15)	0.056 (2)	0.0059 (16)	−0.0117 (18)	0.0060 (14)
C5	0.0344 (16)	0.0284 (14)	0.0346 (14)	0.0049 (12)	0.0053 (11)	0.0003 (11)
C6	0.046 (2)	0.0334 (16)	0.0474 (17)	0.0078 (15)	−0.0082 (14)	0.0083 (13)
C7	0.0440 (19)	0.0258 (14)	0.0479 (17)	0.0042 (13)	−0.0005 (14)	0.0078 (12)
C8	0.054 (2)	0.0432 (19)	0.0512 (19)	0.0102 (17)	−0.0090 (16)	0.0002 (15)
C9	0.051 (2)	0.055 (2)	0.0496 (19)	0.0078 (19)	−0.0028 (16)	−0.0081 (16)
C10	0.048 (2)	0.0325 (16)	0.0457 (17)	0.0038 (14)	0.0073 (14)	0.0013 (13)
C11	0.064 (3)	0.089 (4)	0.078 (3)	0.014 (3)	0.034 (2)	0.004 (3)

### Geometric parameters (Å, °)

**Table:**

Tb1—O2^i^	2.245 (2)	N1—C10	1.335 (4)
Tb1—O1	2.275 (2)	N1—C8	1.388 (5)
Tb1—O12	2.385 (3)	N1—C5	1.433 (4)
Tb1—O3	2.441 (3)	N2—C10	1.323 (5)
Tb1—O6	2.461 (2)	N2—C9	1.342 (6)
Tb1—O4	2.464 (3)	N2—H2N	0.80 (5)
Tb1—O10	2.509 (3)	C1—C2	1.502 (4)
Tb1—O7	2.529 (3)	C2—C3	1.379 (5)
Tb1—O9	2.553 (3)	C2—C7	1.380 (4)
Tb1—N3	2.855 (3)	C3—C4	1.382 (5)
Tb1—N4	2.910 (3)	C3—H3	0.9300
O1—C1	1.242 (4)	C4—C5	1.371 (5)
O2—C1	1.248 (4)	C4—H4	0.9300
O2—Tb1^i^	2.245 (2)	C5—C6	1.378 (5)
O3—N3	1.253 (5)	C6—C7	1.380 (4)
O4—N3	1.248 (4)	C6—H6	0.9300
O5—N3	1.209 (5)	C7—H7	0.9300
O6—N4	1.274 (5)	C8—C9	1.340 (5)
O7—N4	1.252 (5)	C8—H8	0.9300
O8—N4	1.216 (4)	C9—H9	0.9300
O9—N5	1.251 (4)	C10—H10	0.9300
O10—N5	1.261 (4)	C11—H11A	0.9600
O11—N5	1.203 (4)	C11—H11B	0.9600
O12—C11	1.415 (6)	C11—H11C	0.9600
O12—H1O	0.8147		
			
O2^i^—Tb1—O1	93.66 (9)	N5—O10—Tb1	98.07 (18)
O2^i^—Tb1—O12	81.77 (11)	C11—O12—Tb1	130.4 (3)
O1—Tb1—O12	81.94 (10)	C11—O12—H1O	112.9
O2^i^—Tb1—O3	80.85 (11)	Tb1—O12—H1O	115.1
O1—Tb1—O3	125.28 (10)	C10—N1—C8	107.5 (3)
O12—Tb1—O3	148.43 (10)	C10—N1—C5	125.2 (3)
O2^i^—Tb1—O6	152.13 (11)	C8—N1—C5	127.3 (3)
O1—Tb1—O6	81.95 (9)	C10—N2—C9	109.9 (3)
O12—Tb1—O6	124.33 (10)	C10—N2—H2N	126 (4)
O3—Tb1—O6	79.46 (10)	C9—N2—H2N	123 (3)
O2^i^—Tb1—O4	82.58 (10)	O5—N3—O4	122.5 (4)
O1—Tb1—O4	73.61 (10)	O5—N3—O3	120.1 (4)
O12—Tb1—O4	149.92 (11)	O4—N3—O3	117.4 (3)
O3—Tb1—O4	51.67 (10)	O5—N3—Tb1	175.0 (3)
O6—Tb1—O4	69.74 (9)	O4—N3—Tb1	59.30 (18)
O2^i^—Tb1—O10	76.79 (9)	O3—N3—Tb1	58.31 (19)
O1—Tb1—O10	153.55 (10)	O8—N4—O7	122.8 (4)
O12—Tb1—O10	72.39 (10)	O8—N4—O6	120.4 (4)
O3—Tb1—O10	78.08 (10)	O7—N4—O6	116.8 (3)
O6—Tb1—O10	117.71 (8)	O8—N4—Tb1	174.9 (4)
O4—Tb1—O10	128.09 (10)	O7—N4—Tb1	60.03 (17)
O2^i^—Tb1—O7	154.61 (11)	O6—N4—Tb1	57.02 (15)
O1—Tb1—O7	77.57 (10)	O11—N5—O9	122.2 (3)
O12—Tb1—O7	73.48 (10)	O11—N5—O10	121.9 (3)
O3—Tb1—O7	123.72 (10)	O9—N5—O10	115.9 (3)
O6—Tb1—O7	51.06 (10)	O1—C1—O2	124.1 (3)
O4—Tb1—O7	116.66 (10)	O1—C1—C2	118.3 (3)
O10—Tb1—O7	100.48 (10)	O2—C1—C2	117.6 (3)
O2^i^—Tb1—O9	123.29 (9)	C3—C2—C7	119.3 (3)
O1—Tb1—O9	142.55 (9)	C3—C2—C1	120.5 (3)
O12—Tb1—O9	96.38 (12)	C7—C2—C1	120.2 (3)
O3—Tb1—O9	71.99 (11)	C2—C3—C4	120.3 (3)
O6—Tb1—O9	68.14 (9)	C2—C3—H3	119.9
O4—Tb1—O9	113.68 (11)	C4—C3—H3	119.9
O10—Tb1—O9	49.74 (8)	C5—C4—C3	119.8 (3)
O7—Tb1—O9	66.28 (11)	C5—C4—H4	120.1
O2^i^—Tb1—N3	82.04 (11)	C3—C4—H4	120.1
O1—Tb1—N3	99.40 (10)	C4—C5—C6	120.6 (3)
O12—Tb1—N3	163.80 (11)	C4—C5—N1	120.6 (3)
O3—Tb1—N3	25.90 (10)	C6—C5—N1	118.8 (3)
O6—Tb1—N3	71.67 (10)	C5—C6—C7	119.3 (3)
O4—Tb1—N3	25.83 (10)	C5—C6—H6	120.4
O10—Tb1—N3	103.51 (11)	C7—C6—H6	120.4
O7—Tb1—N3	122.65 (10)	C6—C7—C2	120.7 (3)
O9—Tb1—N3	92.25 (12)	C6—C7—H7	119.7
O2^i^—Tb1—N4	173.59 (9)	C2—C7—H7	119.7
O1—Tb1—N4	80.14 (9)	C9—C8—N1	107.0 (3)
O12—Tb1—N4	98.87 (11)	C9—C8—H8	126.5
O3—Tb1—N4	101.31 (11)	N1—C8—H8	126.5
O6—Tb1—N4	25.75 (10)	C8—C9—N2	107.6 (3)
O4—Tb1—N4	94.00 (10)	C8—C9—H9	126.2
O10—Tb1—N4	109.53 (8)	N2—C9—H9	126.2
O7—Tb1—N4	25.40 (10)	N2—C10—N1	108.0 (3)
O9—Tb1—N4	63.06 (9)	N2—C10—H10	126.0
N3—Tb1—N4	97.26 (11)	N1—C10—H10	126.0
C1—O1—Tb1	143.1 (2)	O12—C11—H11A	109.5
C1—O2—Tb1^i^	159.8 (2)	O12—C11—H11B	109.5
N3—O3—Tb1	95.8 (2)	H11A—C11—H11B	109.5
N3—O4—Tb1	94.9 (2)	O12—C11—H11C	109.5
N4—O6—Tb1	97.2 (2)	H11A—C11—H11C	109.5
N4—O7—Tb1	94.6 (2)	H11B—C11—H11C	109.5
N5—O9—Tb1	96.2 (2)		

Symmetry codes: (i) −*x*+1, −*y*+1, −*z*+2.

### Hydrogen-bond geometry (Å, °)

**Table:**

*D*—H···*A*	*D*—H	H···*A*	*D*···*A*	*D*—H···*A*
O12—H1O···O10^ii^	0.81	2.15	2.960 (4)	173
N2—H2N···O6^iii^	0.80 (5)	2.00 (5)	2.779 (4)	167 (5)

Symmetry codes: (ii) −*x*+1, −*y*+2, −*z*+2; (iii) −*x*, −*y*, −*z*+1.
